# Trazodone Loaded Lipid Core Poly (ε-caprolactone) Nanocapsules: Development, Characterization and *in Vivo* Antidepressant Effect Evaluation

**DOI:** 10.1038/s41598-020-58803-z

**Published:** 2020-02-06

**Authors:** Nahla Elhesaisy, Shady Swidan

**Affiliations:** 1 Department of Pharmaceutics, Faculty of Pharmacy, The British University in Egypt, El-Sherouk City, Cairo 11837 Egypt; 2 The Center for Drug Research and Development (CDRD), The British University in Egypt, El-Sherouk city, Cairo 11837 Egypt

**Keywords:** Psychology, Medical research

## Abstract

Trazodone hydrochloride (TRH) is a lipophilic drug which is used effectively as an antidepressant. Its poor solubility and short half-life represent an obstacle for its successful use. Nanocapsules with biodegradable polymeric shell are successful drug delivery systems for controlling the release of drugs. To enhance the entrapment of lipophilic drugs, oils can be added forming a lipophilic core in which the drug is more soluble. The aim of this study was to enhance the efficacy of TRH and prolong its action by formulating it into lipid core polymeric shell nanocapsules. Nanocapules were prepared using nanoprecipitation technique. All prepared formulations were in nano size range and negatively charged. The TRH entrapment efficiency (EE%) in lipid core nanocapsules was up to 74.8 ± 0.5% when using Labrafac lipophile as a lipid core compared to only 55.7 ± 0.9% in lipid free polymeric nanospheres. Controlled TRH release was achieved for all prepared formulations. Forced swim test results indicated the significant enhancement of antidepressant effect of the selected TRH loaded Labrafac lipophile core nanocapsules formulation compared to control and TRH dispersion in phosphate buffer. It is concluded that lipid core nanocapsules is a promising carrier for the enhancement of TRH efficacy.

## Introduction

**Figure 1:**
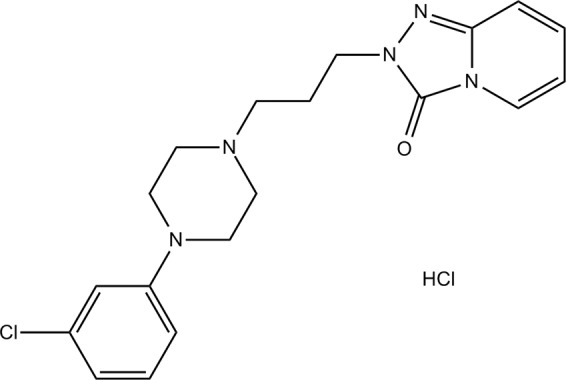
Chemical structure of Trazodone HCl.

Depression is one of the most common, chronic and debilitating psychological disorders that may lead eventually to patient suicide^1^. It is characterized by many symptoms like feeling hopelessness or inappropriate guilt, low energy, change in sleep, change in appetite and recurrent thoughts of suicide. Its consequences not only affect the patient, but also affect negatively the whole society. The financial consequences of this disease are tremendous and cause an overwhelming burden on the society. This burden is due to the dramatically decrease in productivity and absenteeism in the workplace. Depression mainly occurred due to the deficiency of both serotonin and norepinephrine neurotransmitters in the brain^2^. Trazodone hydrochloride (TRH) is one of the most potent drugs used for the treatment of depression (Fig. 1). It is a triazolopyridine derivative with antidepressant effect^3^. It is thought that the mechanism of action of TRH is due to its activity at 5-HT1, 5-HT2 serotonergic receptors. It also acts on poorly blocking serotonin reuptake and selectively blocking presynaptic receptors. In addition, TRH blocks alpha-2 adrenoceptors^4^. TRH possesses unique properties compared to other antidepressants. TRH causes fewer anticholinergic side effects compared to the tricyclic antidepressant. It also has an activity against anxiety which is concomitant to depressed patients. Several studies showed that at therapeutic doses TRH is less likely to cause cardiotoxicity than imipramine. TRH also does not result in neurologic side effects and seems to be well tolerated by elderly^5^. Despite all of these unique properties of TRH, it suffers from some drawbacks that negatively affects its activity. It is a small molecule of molar mass 408.33 g/mol^6^ and it has high lipophilic nature^7^. TRH has a very short half-life about 4.1 h^8^. This leads to frequent administration of TRH which decreases depression patients’ compliance and their adherence to the treatment regimen. Also hydrophobic small molecule drugs suffer from low water solubility and wide tissue distribution profile which may lead to very serious side effects after administration of free drug^9^. Nanotechnology has become one of the main technologies of the 21^st^ century as a result of its revolution in multiple fields like medical, food and pharmaceutical one^10^. Polymeric nanoparticles have attracted attention recently as delivery vesicles for biomedical application. This is due to its unique advantages in targeting, enhancing drugs’ permeation and making controlled release action from the drugs^11,12^. Nanocapsules are nano-sized carrier with two main parts: the inner part is called “core” which is oily in nature while the outer part is a thin polymer shell^13^. Drugs which are encapsulated in nanocapsules not only protected from exterior environment, but also have a facilitated way for controlling their release^14^. One of the most special advantage of nanocapsules as drug delivery system is that it has the ability to successfully encapsulate both hydrophilic and lipophilic drugs according to the nature of its core^15^. Lipid core nanocapsules possess several advantages: they have an excellent ability in preventing the drugs from degradation, they also have high drug loading capacity and reduced burst release^16^. The most commonly used polymers for forming the shell are the biocompatible and biodegradable one like poly lactic acid, poly(lactide-co-glycolide and poly(Ɛ-caprolactone) (PCL)^17^. PCL has promising characteristics in addition to being biocompatible and biodegradable. Elasticity with tensile strength, safety, cytocompatibility and long term degradation are also unique advantages of PCL. Long term degradation property of PCL has been exploited intelligently in several formulations for controlled drug delivery^18^. There are many oils which can represent the lipid core for the lipid core nanocapsules. Long chain oils such as oleic acid are commonly used. While the most commonly used oils forming the core of nanocapsules are triglycerides^19^. Caprylic/capric triglyceride are the most frequent triglycerides used in the lipid core nanocapsules^20–22^. Different methods were used to prepare the nanocapsules, but the most common one is the nanoprecipitation method. Nanoprecipitation method is also called interfacial deposition or solvent displacement method. It is one of the earliest developed methods for encapsulating drugs and it is mainly used for encapsulating hydrophobic drugs^23^. Nanoprecipitation method has many advantages, for example: it is a simple method that can be scaled up easily, it does not need high energy input or large amounts of toxic solvents. The obtained particles from this method are in submicron sizes with narrow size distribution^24^. By using such a simple technique, the aim of this study was to evaluate the efficacy and sustain the effect of TRH by formulation of lipid core PCL shell TRH loaded nanocapsules. This was done by both *in vitro* tests and the assessment of its pharmacological action *in vivo* using forced swim test (FST). It also aims to compare and evaluate the use of different oils for improved entrapment of the drug inside the core of the nanocapsules. Two caprylic/capric (medium chain) triglyceride of widely different hydrophilic lipophilic balance values (HLB) were used, and oleic acid is an example of long chain fatty acids that was also used as lipid core. To our knowledge, except for the patent by Benita *et al*., TRH was not formulated into nanocapsules before^25^. The *in vivo* pharmacological action of TRH in the nanocapsules will be evaluated for the first time.

## Results and Discussion

### Determination of solubility of TRH in the core oil

Trazodone is a hydrophobic drug, which can be protonated; its aqueous solubility depends upon the pH. The log P of TRH is 3.13. The solubility of the free base TRH was experimentally determined to be 0.176 mg/ml at pH 11.5^26^ and 0.081 mg/ml at pH 10 at 20 °C^27^, additionally its solubility was calculated to be 0.29 mg/ml^28^. The apparent solubility of the HCl salt of TRH was reported to be 38.5 mg/ml^29^ and 22 mg/ml at a pH of 5.0^30^. The n-octanol water partition coefficient of TRH was found to be 63.3 at pH 7.4^31^. It is clear that the inclusion of this drug to oil core nanocapsule will increase its loading and entrapment into the nanocapsules. The solubility of TRH in labrafac lipophile, Miglyol 812 and oleic acid was 1.025 mg/ml, 0.758 and 0.965 mg/ml respectively. This may be explained according to the HLB of the oil in which the TRH is dissolved. The HLB of Miglyol 812 is 15, which indicates higher hydrophilicity compared to the other oils which both have HLB value of 1. The lipophilicity of the latter oils allow higher amount of the drug to be dissolved in. The TRH solubility is higer in labrafac lipophile than in the oleic acid. Although both have the same HLB, the higher solubility in labrafac lipophile is attributed to the chain length of the oil. The shorter chain triglycerides have more polar groups per unit mass than longer chain molecules, so there is dipole–dipole interactions between polar groups on the oil and TRH molecules which may increase the solubilization of the drug^32^.

### Charecteriation of the prepared TRH loaded nanospheres and nanocapsules

#### Particle size and zeta potential analysis

All prepared formulations were in the nanorange, the oil free nanospheres had particle size (PS) of 118.4 ± 1.8 nm. The lipid core nanocapsules size ranged from 133.5 ± 2.1to 172.4 ± 2.2 nm. As seen from these results shown in Table (1), the inclusion of the oil increases the nanocapsule size and from our preliminary studies results, the presence of the TRH had a major effect on the size as well. The smallest PS of lipid core nanocapsules was achieved in F2 where labrafac lipophile is the core oil, while the largest PS was in the Miglyol 812 containing nanocapsules. The small PS of the polymeric nanosphere without oil in the core was in agreement with Stella *et al*., who prepared gemcitabine lipophilic derivatives into polycyanoacrylate nanospheres and nanocapsules with Miglyol 812 in the core. They found that the mean size of the nanospheres formulations is significantly smaller than that of the oil core nanocapsules^33^. Heurtault *et al*., and Huynh *et al*., found that increasing the concentration of oil in the nanocapsules core leads to increasing the particle size of the nanovesicles^34,35^. The polydispersity index (PDI) is a dimensionless value describing the size distribution in colloids, it is an important parameter to ensure size homogenity. Its value varies from 0.0 to 1.0, the smaller the PDI value – closer to zero – the more homogenous the vesicles will be. As shown in Table (1), F1, F2, and F3 had PDI values of 0.19 ± 0.03, 0.29 ± 0.01 and 0.28 ± 0.01 respectively. These formulations showed acceptable size distrbution as PDI is <0.3. The widest size distribution was found in F4 with PDI 0.33 ± 0.02. It is clear that TRH loaded nanospheres showed more homogenous size distribution compared to oil core nanocapsules. Stella and colleagues should also that the average PDI of the drug loaded nanospheres was much lower than that of the drug loaded oil core nanocapsules^33^.

**Table 1:** Particle size (PS), polydispersity index (PDI), zeta potential (ZP) of the prepared TRH loaded nanocapsules.

Form.	PS (nm)	PDI	ZP (mV)
F1	118.4 ± 1.8	0.19 ± 0.03	−19.4 ± 0.5
F2	133.5 ± 2.1	0.29 ± 0.01	−21.9 ± 0.9
F3	172.4 ± 2.2	0.28 ± 0.01	−21.4 ± 0.3
F4	146.7 ± 2.1	0.33 ± 0.02	−23.3 ± 0.2

Surface charge is a good indication of stability of colloidal systems, higher zeta potential (ZP) values ensures high stability of the colloidal dispersions. Ideally, the stability of nanoparticles is specified by the balance between the forces of attraction and repulsion. This is the main factor affecting the stability in the absence of steric effect. The strong repulsive forces help in the prevention of particle agglomeration. The most stable nanoparticle suspension is achieved when the ZP is greater than ± 30 mV. While, if there is a combined steric and electrostatic stabilization, a minimum ZP of ± 20 mV is sufficient for long term stability^36^. As shown in Table (1), all prepared formulations showed relatively high ZP. They ranged between −19.4 ± 0.5 to −23.3 ± 0.2 mV. Because the stabilizers used are non-ionic surfactants (span 60 and poloxamer 188), the negative value is expected not to be too high. Similar results were obtained by Lboutounne *et al*.^37^, who prepared poly(ε-caprolactone) without inclusion of lipid core and the ZP value was − 20.9 mV. Due to presence of oil in the core, a slight increase in the ZP was observed in F2,F3,F4 than in F1. Oleic acid has strong negative charge, but due to the inclusion of the oil into the core of the nanocapsules, the oleic acid resulted in slight increase in ZP. The nonionic surfactants can participate in the stability of the nanocapsules due to their steric effect. As mentioned by Lourenco *et al*., the polymeric nanocapsule can be sterically stabilized by the use of poloxamers and polysorbates^38^.

#### Encapsulation efficiency

**Figure 2:**
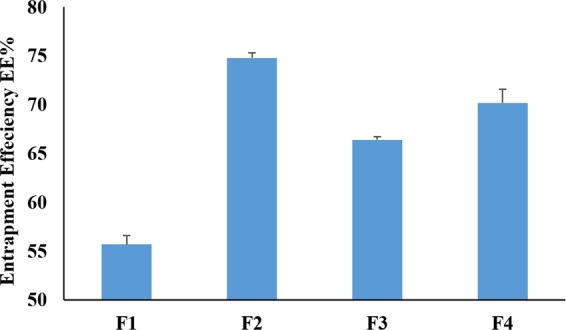
EE% of TRH in the prepared formulations.

To investigate the influence of the lipid type on the EE% of the TRH in the nanocapsules, different formulations with different lipids were prepared and compared to polymeric nanocapsules containing no oil in their core. The EE% of the prepared formulations are illustrated in Fig. (2). As seen from the figure, higher EE% results were achieved by the addition of the lipids to the core of the nanocapsule. As F1 had only 55.7 ± 0.9% of TRH entrapped in the nanospheres, the EE% of the lipid core nanocapsules were 74.8 ± 2.1%, 66.4 ± 0.5% and 70.2 ± 1.2% for formulations F2, F3 and F4 respectively. Nanocapsules containing Labrafc liophile in the core showed the highest EE%, followed by F4 containing oleic acid, then F3 with Miglyol 812 which showed the least EE% among the prepared lipid core nanapcasules. This was in complete agreement with the solubility study conducted in this work. TRH is a highly lipophilic drug which pass the blood brain barrier easily, so it was expected to have poor loading to the PCL nanospheres. The lipophilic drugs tend to concentrate away from the polymeric shell and concentrate in the core^39^. So by adding the lipid to the core higher EE% is expected to be obtained. Different factors control the extent of TRH entrapment to the lipid core nanocapsule such as the solubility of TRH in the lipid forming the core of the nanocapsules and the partition coefficient of the drug between the lipid core and the aqueous medium. Dalencon and co-workers studied the solubility of Rifabutine and Atovaquone in different lipids. They found that the highest solubility of the drugs was in benzyl benzoate which achieved much higher EE% for both drugs compared to decreased EE% in the nanocapsules where the lipids showed poorer drug solubilities^40^. Stella and colleagues studied the effect of the difference in partition of different drugs between Miglyol 812 and water. They found that the stability of the entrapped drug within the inner core of the nanocapsules depends on the transfer rate by diffusion in the aqueous medium. This is crucially dependent on the partition coefficient of the drug between the nanoparticle core and the aqueous phase; it was found that the higher the partition coefficient, the slower was the transfer rate^33^.

#### Study of the *in vitro* release of TRH from the prepared nanospheres and nanocapsules

**Figure 3:**
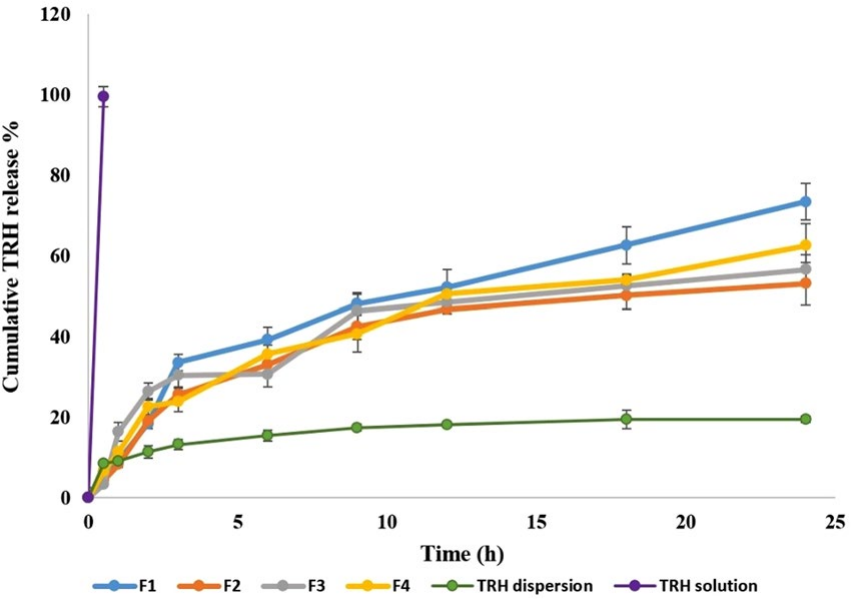
*In vitro* TRH release from different prepared formulations compared to TRH dispersion in phosphate buffer pH 7.4 and TRH solution in phosphate buffer pH 7.4 solubilized with Tween 80.

The *in vitro* release study was done using the dialysis membrane technique. TRH release from all formulations was studied and compared with the release of the pure drug from both TRH dispersion in phosphate buffer pH 7.4 and solubilized TRH in Tween 80 phosphate buffer pH 7.4 solution. The TRH release data is illustrated in Fig. (3). All prepared nanospheres and oil core nanocapsules showed controlled release compared to immediate complete release from TRH solubilized solution in tween 80 and poor incomplete release from TRH dispersion in phosphate buffer. After 24 h, the release from formulations F1, F2, F3 and F4 was 73.4 ± 4.5%, 53.1 ± 5.3%, 56.6 ± 3.6, 62.5 ± 5.5 respectively. This was compared to about 100% of TRH released from TRH solubilized solution after 30 min. showing complete burst release of the drug through the dialysis membrane. On the other hand, TRH dispersion released only 19.5 ± 0.8%; this might be due to the poor solubility of TRH which allowed only very small amount of the drug to be released which is determined by the saturation solubility of TRH in phosphate buffer. By comparing the oil free nanospheres with the three lipid core nanocapsule formulations, it is clear that the presence of TRH solubilized form in a lipophilic environment was reflected on the controlled manner of the release. Jäger *et al*., suggested that the presence of the oil in the core of the nanocapsules delayed the release of Indomethacin ester and its release was slow and time dependent. This group studied also the effect of the viscosity of the core oil and its effect on the release of the drug. They found that when the lipid core composed of caprylic/capric triglyceride increased in concentration, it reflects a big reduction in the viscosity of the nanocapsule dispersion. This in turn caused an increase in the half-lives of the Indomethacin ester release which referred to delayed effect with decreasing viscosity^41^. The addition of lipid to the core of the nanocapsule allows the hydrophobic drugs to be more concentrated in the core so the burst release due to erosion of the nanocapsule shell is minimized^39^. The higher release rate of TRH from F1 might also be due to the smaller PS because of the absence of the lipid core. This led to increase in the surface area of the nanocapsules which increases the release rate of the TRH^41^.

#### Kinetic study of the *in vitro* TRH release

The release kinetics of the prepared formulations was evaluated by fitting the obtained *in vitro* TRH release to zero order, first order, Higuchi diffusion and Hixon Crowel models. The correlation coefficient (r) was calculated for each model as shown in Table (2). As seen from the table, oil free nanospheres and lipid core nanocapsules are best fitted to Higuchi diffusion model. In this model, the drug released from the nanocapsule after the degradation of the shell is controlled by the diffusion mechanism rather than matrix erosion mechanism. Similar results obtained by Derakhshandeh and co-workers who encapsulated 9-nitrocamptothecin into PLGA nanoparticles. They found that the best fitted model for the *in vitro* release of the drug was Higuchi model and they concluded that the release of the drug was by diffusion mechanism^42^. The half life of TRH obtained by applying Higuchi model was 10.19 h for the PCL nanosphere where the drug is dispersed in the polymer. The half lives obtained in F2, F3 and F4 were 17.13, 17.11 and 14.32 h respectively. The half life values indicated that the most controlled release manner was obtained by F2 which contains Labrafac lipophile as the lipid core. The overall *in vitro* release data showed that nanoencapsulation of TRH into nanocapsules offered controlled time dependent release. It also indicated that the formulation of the lipid core nanocapsules offered more sustained release of TRH to the release medium.

**Table 2:** The calculated correlation coefficient (r) obtained from fitting the TRH *in vitro* release data to different kinetic models.

Formulation No.	Correlation coefficient (r)			
	Zero order	First order	Higuchi Diffusion	Hixon-Crowel
F1	0.936	0.032	0.988	0.970
F2	0.896	0.155	0.976	0.919
F3	0.876	0.149	0.963	0.908
F4	0.933	0.113	0.991	0.959

**Figure 4:**
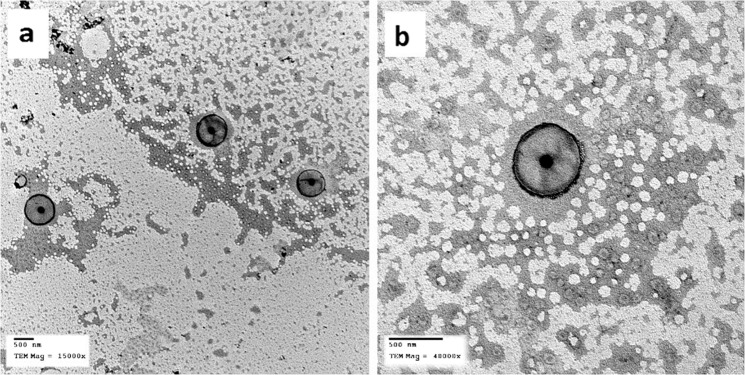
TEM photographs of the TRH loaded nanocapsules containing Labrafac Lipophile as a core lipid (F2) (**a**) Magnification power 15000X, (**b**) Magnification power 40000X.

Morphological evaluation of the selected TRH nanocapsule formulation. According to the EE% and the release data, F2 was selected to be evaluated *in vivo*. The highest EE% and the most controlled prolonged TRH release pattern made F2 a promising system for enhancing the antidepressant activity of TRH. Before the *in vivo* experiment was done, F2 was morphologically studied using TEM. The morphology of F2 nanocapsules is shown in Fig. (4) with two different magnification powers. As seen from the figure, spherical capsules with homogenous size were formed, no agglomerations can be seen. The capsular shape and structure shows the polymer membrane as a line surrounding an oily core. The mean PS of the nanocapsule observed by TEM is comparable with the mean size obtained using the Malvern Zetasizer. Similar shapes were obtained in formulation of different core shell nanocapsules as mentioned by Jovanovic *et al*., and Li *et al*.^43,44^.

### Assessment of the pharmacological action

#### Forced swim test

**Figure 5:**
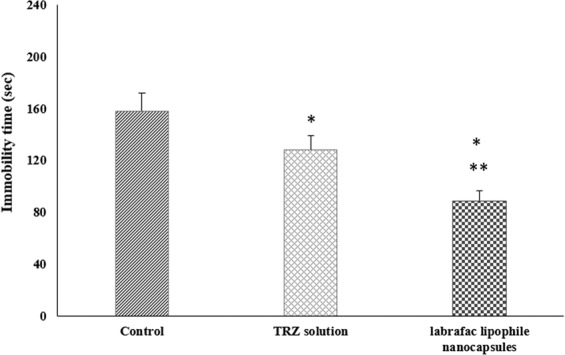
Immobility time of forced swim test for TRH dose equivalent to 5 mg/kg (*P < 0.05). *Is significant difference in groups compared to control group. **Is significant difference in groups compared to TRH dispersion group.

The FST is a well-established model for predicting the clinical efficacy of antidepressant drugs^45,46^. It has been described as making of a situation in which “behavioral despair” is induced; in such situation, the animal loses hope to escape the stressful environment^47^. In this test, the time spent by each mouse in mobility during a 4 min. period is measured. The total mobility time is then subtracted from the 240 sec. of test time and is then stated as the immobility time^48^. As seen from the results shown in Fig. (5), the mean immobility time for the control group was 158 ± 15 sec. (n = 8). The group injected with trazodone dispersion in phosphate buffer pH 7.4 had a mean immobility time of 128 ± 12 sec. (n = 8). The shortest immobility time was observed in the F2 nanocapsule group with a mean immobility time of 88 ± 8 sec. (n = 8). There was significant decrease in immobility time for the TRH dispersion and F2, compared to the control group which received the vehicle only (P < 0.05). The group treated with TRH dispersion showed a decrease in the immobility time by 18.98%. There was also a significant difference in the immobility time between the F2 nanocapsules in which the core is labrafac lipophile and the TRH dispersion (P < 0.05). The third group injected with F2 showed a decrease in the immobility time by 44.06% compared to the control group, while the percentage decrease compared to the TRH was also significantly high and reached about 30.95%. As expected from the *in vitro* results, the dispersion of the drug into lipophilic core resulted in controlled release manner of TRH, this could increase the bioavailability of the drug compared to the drug solution. TRH encapsulation into nanocapsules enhance its absorption and prolongs TRH circulation time in the blood, this might explain the improvement of the pharmacological effect of encapsulated TRH compared to the unencapsulated drug. Another cause of the improvement of the welling to escape behavior of the mice in the F2 TRH nanocapsule is that the drug is entrapped in a lipophilic environment formed by the labrafac lipophile in the core of the nanocapsules. This lipid core not only allows higher entrapment of TRH, but it also prevents its precipitation during the preparation and decrease the burst release of TRH^49^.

## Conclusion

In the present study, TRH nanospheres and TRH lipid-core nanocapsules were prepared successfully using nanoprecipitation method. Different formulations were prepared using different types of lipid forming the cores of the nanocapsule. All the prepared formulations were in nano range with negative ZP. The formation of lipid core in the nanocapsules showed marked increase in TRH EE%. The *in vitro* release results showed that controlled slow release was achieved from the nanocapsules and the nanospheres formulations in comparison with TRH dispersion in phosphate buffer. The highest entrapment efficiency was for F2 which contains labrafac lipophile lipid core. TEM showed that nanocapsules were spherical in shape and uniform in size. The *in vivo* FST supported the *in vitro* results as the immobility time for the swimming mice were decreased which indicated significantly enhanced antidepressant effect of the selected formulation F2 compared to control and TRH dispersion in phosphate buffer pH 7.4. Finally, it can be concluded that lipid core PCL shell nanocapsules are promising carrier for controlling the TRH release and enhancement of efficacy of TRH *in vitro* and *in vivo*.

## Materials and Methods

### Materials

Trazodone hydrochloride, miglyol 812 and Poloxamer 188 were kind gifts from the Egyptian International Pharmaceutical Industries Company (EPICO), Cairo, Egypt. Span 60 (Sorbitan Monostearate) was purchased from Oxford-Laboratory chemicals, Mumbai India. Labrafac Lipophile WL 1349 (Medium chain triglyceride) was supplied by Gattefosse Saint-Priest, France. poly(ε-caprolactone) (Mwt. 14000) was purchased from Sigma-Aldrich, St.Louis, USA. Oleic acid was purchased from Chemajet, Cairo, Egypt. Acetone was purchased from Al Ahram laboratory chemicals company Cairo, Egypt. Sodium hydroxide was purchased from Lobechem, Delhi, India, while potassium dihydrogen orthophosphate was purchased from El Nasr pharmaceutical chemicals Company, Cairo, Egypt. All other solvents and chemicals are of pharmaceutical grade and were used with no further modifications.

### Methods

#### Determination of solubility of TRH in the core oil

The solubility of TRH in the core oils were determined using the shaking method as described by Setthacheewakul *et al*., with modifications^50^. An excess amount of the TRH was dissolved in 1 ml of each oil in a cap vial. Then the sealed vials were sonicated in sonicating water bath (Elma Sonic, S. 30, Elma Schmidbauer GmbH, Germany) for 10 min. to insure proper mixing of the drug with each oil. The mixture was then shaken in a shaking water bath (Wise^®^ bath – water bath, Wised B, Germany) for 48 hours at 100 rpm at room temperature. The mixture was centrifuged at 15000 rpm for 10 min. at 4 °C in cooling centrifuge (Centurion Scientific Ltd., UK). The supernatant was then diluted to a suitable dilution using a mixture of acetone and methanol at 1:1 ratio (v/v)^51^. To zero the spectrophotometer for each oil, a cuvette containing each pure oil was used as a reference cell. The λ_max_ used in the measurements was different for each oil and ranged from 246 to 253 nm. A calibration curve was constructed for each oil phase by measuring the absorbance against TRH concentrations in µg/ml. The test was done in triplicate and the mean was reported.

#### Preparation of TRH loaded nanospheres and nanocapsules

Lipid core TRH nanocapsules in addition to oil free nanospheres were prepared using nanoprecipitation technique as described by Ünal *et al*., with slight modifications^52^. Briefly, The aqueous phase was prepared by dissolving poloxamer 188 (77 mg) in 30 ml water. The organic phase of F1 was prepared by dissolving TRH (50 mg), PCL (100 mg), in 15 ml acetone. For F2, F3, F4, span 60 (39 mg), and 158 µl of the Labrafac lipophil, Miglyol 812 and oleic acid respectively were added to the preiviously described acetone solution. Under moderate magnetic stirring, the organic phase was injected into the aqueous phase using a syringe with a controlled adequate rate at room temperature till white bluish opalescent dispersion was formed. The obtained dispersion is then concentrated to a total volume of 10 ml by evaporation of the solvents under reduced pressure at 40 °C using rotary evaporator (BÜCHI Rotavapor R-114, BÜCHI, Lausanne, Switzerland). The compositions of all prepared formulations were listed in Table (3).

**Table 3:** Composition of the prepared TRH loaded nanospheres (F1) and oil core nanocapsules.

	Polymer	Lipid core	Stabilizers
F1	PCL	none	Poloxamer 188
F2	PCL	Labrafac lipophile	Span 60 + Poloxamer 188
F3	PCL	Miglyol 812	Span 60 + Poloxamer 188
F4	PCL	Oleic acid	Span 60 + Poloxamer 188

*TRH conc. was adjusted to 5 mg/ml in all formulations.

### Charecterization of the prepared TRH loaded nanospheres and nanocapsules

#### Particle size and zeta potential analysis

The mean PS of TRH nanocapsules and PDI were determined using dynamic light scattering technique. The measurements were done using Zetasizer Nano ZS (Malvern Instruments, Malvern, UK). Surface charge is indicated by the ZP on the TRH nanocapsule surface. It was also determined using the same device by observing the electrophoretic mobility of the nanoparticles in an electrical field. Prior to the particle size measurement, samples were diluted to a suitable dilution with double distilled water and vortexed for 5 sec. to separate any agglomerations. ZP measurements were done without dilution of the sample. The measurements were done in triplicate and the average of PS, PDI and ZP was calculated. Standard deviation (SD) were also calculated.

#### Determination of encapsulation efficiency

Encapsulation Efficiency (EE %) of TRH nanocapsules was calculated using indirect method in which the amount of free non-encapsulated drug is measured as mentioned by Prego *et al*.^53^. Briefly, 500 µl of each prepared formulations was centrifuged using cooling centrifuge at speed of 15,000 rpm at temperature of 4 °C for 60 min. The supernatant was separated and diluted at suitable dilution, then its concentration was measured using UV spectrophotometer (Jenway 6305 spectrophotometer, China) at predetermined λ_max_ 253 nm. This experiment was done in triplicate and both average and SD were measured. The equation used for calculating amount of encapsulated TRH indirectly is:

$${\rm{EE}}\, \% \,=\,\frac{{\rm{total}}\,{\rm{weight}}\,{\rm{of}}\,{\rm{TRH}}-{\rm{weight}}\,{\rm{of}}\,{\rm{TRH}}\,{\rm{in}}\,{\rm{supernatent}}}{{\rm{total}}\,{\rm{weight}}\,{\rm{of}}\,{\rm{TRH}}}\times 100$$

#### *In vitro* TRH release through dialysis bag

Dialysis bag technique was used for determining the amount of *in vitro* TRH release from both nanospheres and lipid core TRH nanocapsules^54^. Dialysis bags used were with molecular weight cut off (12000 to 14000) were soaked for 24 h before use in the release medium. One ml of each prepared formulation was added into a dialysis bag firmly sealed by double-folding on both sides. For the sake of comparison, TRH dispersion in phosphate buffer and solubilized TRH solution in Tween 80 were evaluated with the same method. The dialysis bags containing formulations were immersed in 50 ml of simulated blood release medium phosphate buffer (pH 7.4) in 100 ml beakers. Afterwards, the beakers were placed in shaking water bath for 24 h at 37 ± 0.5 °C and at 50 rpm. At selected time intervals, 1 ml sample was withdrawn from the dialysis medium and subjected to suitable dilution to analyze the concentration of released TRH spectrophotmetrically at λ_max_ 253 nm. Continuous replacements of the withdrawn samples with fresh buffer were done to the dialysis medium, therefore its volume was kept constant during the whole experiment.

#### Kinetic study of the *in vitro* TRH release

The data obtained from *in vitro* TRH release from the prepared nanospheres and nanocapsules was subjected to study using different release kinetic models in order to determine the TRH release mechanism in the phosphate buffer pH 7.4. These models are useful in predicting the *in vivo* bioperformance of the prepared nanoparticles and predict their controlled release mechanism *in vivo*^55^. The models studied were zero order model, first order model, Higuchi diffusion model and Hixon-Crowell model. The half-life was calculated from each model and the best fitted model was selected based on the highest correlation coefficient (r) value.

#### Morphological evaluation of the selected TRH nanocapsule formulation

The morphology of the best formulation after characterization and *in vitro* release was studied using transmission electron microscope (TEM) (JTEM-1010, JEOL^®^, Tokyo, Japan). The method used for TEM measurement was the negative staining method. In brief, one drop of the nanocapsule formulation dispersion was put on a carbon film-covered copper grid. Filter paper was used for the removal of any excess droplets. Then one drop of uranylacetate solution (2% w/v) was added in drops on the grid. The sample was dried by air at room temperature. TEM investigations were done at 74 kV.

### Assessment of the pharmacological action

#### Forced swim test

**Figure 6:**
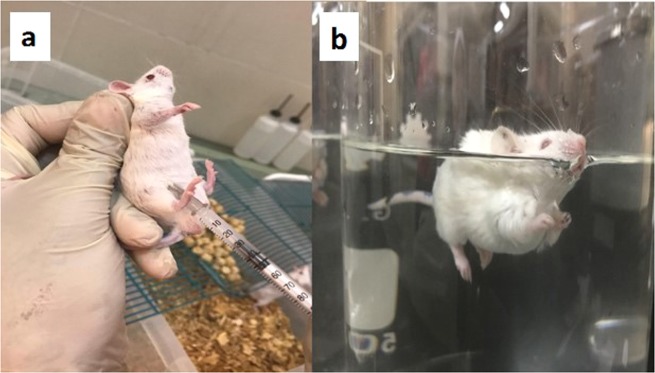
Forced swim test (**a**) intraperitoneal injection, (**b**) immobile mouse during the experiment.

FST is one of the best screening test models for the evaluation of antidepressant activity. It is a reliable and fast model for testing potential antidepressant treatments with strong predictive validity^56^. In this study, FST test was done according to Can *et al*., and De Caro *et al*., with some modifications^48,57^. The procedures employed in this study were reviewed and approved by the ethics committee of the Faculty of Pharmacy, The British University in Egypt. Twenty four male albino mice weighing 40 ± 5 gm were divided into three groups (n = 8). All mice were freely accessed water and food during the whole experiment at room temperature with 12 h light/dark cycle. The mice of the three groups were injected with saline, TRH dispersion or the selected lipid core nanocapsule formulation with the best *in vitro* results with a dose equivalent to 5 mg/kg TRH through the intraperitoneal route (Fig. 6a). Injection period was for five consecutive days. To evaluate the sustained effect of TRH, the test was done after 8 hours of the fifth injection. The mice were individually placed in a glass beaker containing water at room temperature (25 ± 1 °C). The height of the glass beaker is 27 cm, with a diameter of 18 cm and water level was adjusted to 20 cm. This water level allows the prevention of false negative results; although the mice could touch the bottom of the beaker with their tails, but they could not support themselves with their hindlimbs. The movements of each mouse in water were recorded using fixed video camera (DSC-W530, Sony, Japan) for 6 min. and the time that each mice spent on movement was measured. After the test, the mice were removed from the water and dried under lamp before being returned to their cages. The experiment was illuminated by indirect light and all groups were tested on the same day. In the video analysis, the first two min. was excluded due to the fact that most mice are very active at the beginning of the FST and the mobility time was recorded. The total amount of mobility time is then subtracted from the 240 sec. of test time and is then stated as the immobility time^42^. The mobility of the mice is defined according to Belozertseva *et al*., as swimming, rigorous movements with all four legs; paddling, floating with rhythmical simultaneous kicks and occasional pushes off the wall to give speed and direction to the drift. While the immobility is defined as the floating was scored when the animal remained in water with all four limbs motionless, except for occasional alternate movements of paws and tail necessary to prevent sinking and to keep head/nose above water (Fig. 6b)^58^. The time measurement of the mice mobility was done using on-screen stopwatch software (Xnote Stopwatch, dnSoft Research Group). The study was done in triplicate and the mean and SD were calculated.

### Statistical analysis

Statistical analysis of data was done using IBM SPSS^®^ statistics 19 software (SPSS, USA). The analysis was performed by applying one-way analysis of variance (ANOVA) followed by Tukey test. A value of 0.05 was considered statistically significant. All methods were performed in accordance with guidelines and regulations approved by the ethics committee of the Faculty of Pharmacy, The British University in Egypt.
